# Entomovirological Surveillance in Schools: Are They a Source for Arboviral Diseases Transmission?

**DOI:** 10.3390/ijerph18116137

**Published:** 2021-06-06

**Authors:** Juliana Pérez-Pérez, Víctor Hugo Peña-García, Arley Calle-Tobón, Marcela Quimbayo-Forero, Raúl Rojo, Enrique Henao, Talya Shragai, Guillermo Rúa-Uribe

**Affiliations:** 1 Facultad de Medicina, Universidad de Antioquia, Carrera 51D # 62-29 Laboratorio 321, Medellín 050010, Colombia; victorhugopega@gmail.com (V.H.P.-G.); arley.calle@udea.edu.co (A.C.-T.); marceladelpilar45@gmail.com (M.Q.-F.); guillermo.rua@udea.edu.co (G.R.-U.); 2 Centro Administrativo La Alpujarra, Secretaría de Salud de Medellín, Medellín 050015, Colombia; raul.rojo@medellin.gov.co (R.R.); enrique.henao@medellin.gov.co (E.H.); 3 Department of Entomology, Cornell University, Ithaca, NY 14853, USA; tshragai@gmail.com

**Keywords:** arbovirus, *Aedes*, schools, entomological indices, infection rates

## Abstract

Surveillance and control activities for virus-transmitting mosquitoes have primarily focused on dwellings. There is little information about viral circulation in heavily trafficked places such as schools. We collected and analyzed data to assess the presence and prevalence of dengue, chikungunya, and Zika viruses in mosquitoes, and measured *Aedes* indices in schools in Medellín (Colombia) between 2016–2018. In 43.27% of 2632 visits we collected *Aedes* adults, creating 883 pools analyzed by RT-PCR. 14.27% of pools yielded positive for dengue or Zika (infection rates of 1.75–296.29 for *Aedes aegypti*). *Ae. aegypti* was more abundant and had a higher infection rate for all studied diseases. *Aedes* indices varied over time. There was no association between *Aedes* abundance and mosquito infection rates, but the latter did correlate with cases of arboviral disease and climate. Results suggest schools are important sources of arbovirus and health agencies should include these sites in surveillance programs; it is essential to know the source for arboviral diseases transmission and the identification of the most population groups exposed to these diseases to research and developing new strategies.

## 1. Introduction

During the last few decades, the incidence of arboviral diseases has increased worldwide. A total of 390 million dengue cases are estimated every year, 96 million of which show symptoms and about 20,000 are fatal, mostly among children [1,2]. In 2016, more than 2.3 million cases were reported just in the Americas, 1032 of which were fatal [3], and 2019 had the highest number of dengue cases ever recorded in the Americas with more than 3 million reported cases and 1400 registered deaths [3]. Colombia is no stranger to this problem; in 2019, 127,553 cases and 87 confirmed deaths were reported. Cities such as Cali, Medellín, and Bucaramanga have been classified as hyperendemic because there is periodic circulation of all four dengue serotypes and these cities historically have had the highest burden of dengue in Colombia [4]. Although this disease can occur at any age, a study performed in Colombia found that 50.7% of cases were observed in individuals under 19 years old, whereas 49.5% of severe dengue cases were observed in children aged under 14 years old [5].

Recently, Zika and chikungunya have emerged as new arboviruses in the Americas with significant public health, social, and economic impact. After Brazil, Colombia had the highest number of reported Zika cases in South America during the outbreaks that occurred between 2015–2016 [6]. Also, chikungunya virus has caused considerable outbreaks worldwide. In 2015, ~700,000 probable cases were reported in the Americas, most of them (356,079 cases) in Colombia. These arboviruses are primarily transmitted by the bite of *Aedes aegypti* and *Ae. albopictus* mosquitoes, both of which are present in Colombia. Moreover, in the case of Zika, natural infection and experimental transmission have been reported in *Culex* mosquitoes [7,8,9]. Because few vaccines are currently available to prevent the arboviral diseases of public health importance, the prevention and control efforts focus on reducing mosquito populations, and the main prevention strategy is vector control [10,11], which has traditionally focused on people’s dwellings and residential areas [12,13]. However, there are non-residential areas that are highly trafficked and where immature and adult forms of these vectors are often identified [14,15,16,17]. Despite the potential importance of these other locations, there are few studies that have analyzed their role in arboviral transmission. Recently, new strategies for the prevention of disease caused by arboviruses have been developed, including the possible use of mosquito saliva proteins as a universal vaccine against arboviruses; it is very important to know the source for arboviral diseases transmission and the most exposed population to help specialists to research and develop on the relevant vaccines.

In Medellín, the Secretariat of Health (SSM, by its Spanish acronym) implemented a strategy called Healthy Schools, a tool aimed at performing health-promoting activities and preventing arboviral diseases at schools. Healthy Schools includes a vector control program where traditional *Aedes* indices are routinely measured to identify schools with the highest abundance of *Aedes* mosquitoes to then focus control efforts on those locations (SSM 2018). However, multiple studies have shown that there is a weak association between traditional *Aedes* indices and the risk of arboviral transmission [18], and detecting mosquito infection rates for arboviral disease may be a better measure of human risk [19,20]. In this study, we analyzed natural infection with dengue, Zika, and chikungunya viruses in *Ae. aegypti* and *Ae. albopictus* mosquitoes collected in schools in Medellín, as well as the association between infection rates in mosquitoes and traditional *Aedes* indices, some epidemiological (dengue and Zika cases) and climate (temperature, relative humidity, and precipitation) variables. The implications of arbovirus infection in mosquitoes collected in schools are then discussed in the context of disease mitigation in educational establishments.

## 2. Materials and Methods

### 2.1. Study Area

Medellín is in the department of Antioquia, at 75°34′05″ W and 6°13′55″ N and is approximately 376.2 km^2^. Medellín is relatively flat in the city center, which is at 1500 masl (meters above sea level), with a steep rise along the perimeter up to 1800 masl. The climate is subtropical sub-humid with mean temperatures ranging from 16 to 28 °C. The population is around 2.5 million, and both dengue and Zika are routinely reported. Chikungunya cases in Medellín occur on a sporadic basis [21].

### 2.2. Mosquito Collections and Traditional Aedes Indices

**Figure 1:**
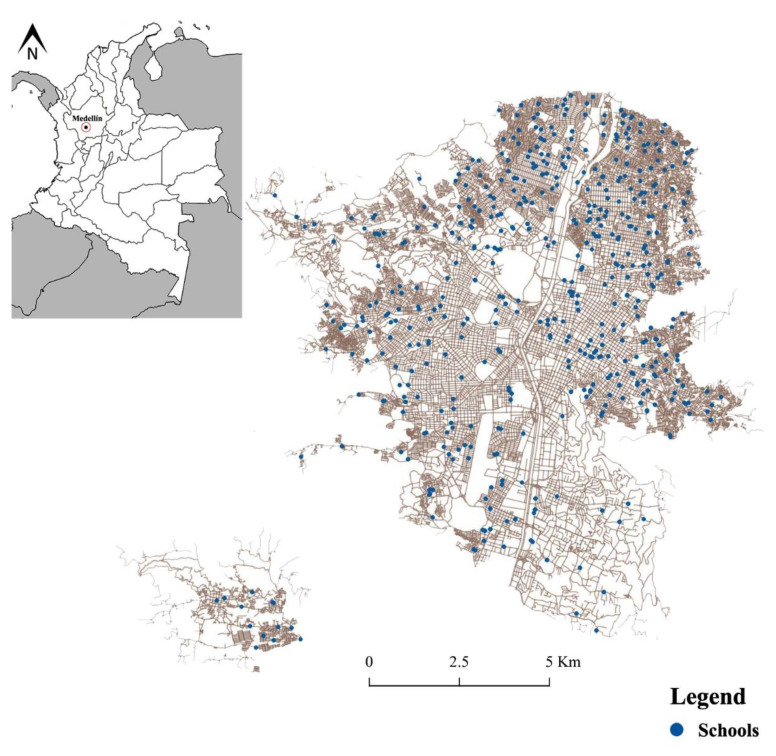
Location of schools in Medellín, Colombia included in this study.

Between January 2016 and December 2018, 2362 inspections were performed to capture adult mosquitoes (Figure 1). Schools included elementary and high; these were chosen for Medellin Health Secretary criteria. Schools were distributed throughout the city. Each visit was performed by a single official, who spent between 45 and 60 min at each institution, depending on its size. An active search was used to collect mosquitoes with a mouth aspirator and an entomological net among common areas such as offices, classrooms, bathrooms, and different humid and dark places, which are the preferred resting places of these vectors [22,23]. Mosquitoes were stored in tubes and transported alive to the Medical Entomology Laboratory of the School of Medicine of Universidad de Antioquia for identification through published keys [24].

The presence and number of potential larval development sites (water-holding containers), such as tires, flower vessels, bottles, trash cans, or gutters, within each school was recorded. All containers and schools were visually assessed for the presence of immature forms and adults to calculate the following traditional *Aedes* indices: Adult Index (AI) (the percentage of schools infested with adults *Aedes*), house index (HI) (the percentage of schools infested with larvae and/or pupae), container index (CI) (the percentage of water-holding containers infested with larvae or pupae), and Breteau index (BI) (the number of positive containers per 100 schools inspected). Containers were counted as positive if they had immature forms of the vector, whereas schools were considered positive if at least one container with immature forms of *Aedes* spp. During 2016, schools were inspected once per trimester, and in 2017 and 2018 schools were visited once a month. We aggregated ento-virological data to a three-month scale to enable comparison to entomological risk indicators. The level of risk was based on three levels of risk for each index, as suggested by the Health Department of Antioquia: low risk (BI < 5%, HI < 4%, CI < 3%), medium risk (BI 5–50%, HI 4–35%, CI 3–20%), and high risk (BI > 50%, HI > 35%, CI > 20%).

### 2.3. Molecular Arbovirus Detection in Mosquitoes

Mosquitoes were divided into pools according to species (*Ae. aegypti* and *Ae. albopictus*) and collection site, each pool contained from 1 to 10 mosquitoes. Mosquitoes captured in 2016 were only tested for dengue virus, and in 2017 and 2018 samples were tested for dengue, chikungunya, and Zika. RNA was obtained with the RNeasy Mini Kit (Qiagen, Germantown, USA commercial kit. Each pool was mechanically macerated in 100 µL of lysis buffer (buffer RLT) and RNA was obtained with the RNeasy Mini Kit (Qiagen) commercial kit according to manufacturer instructions. Viral detection was performed with RT-PCR. Copy DNA (cDNA) was synthesized using 10 µL extracted RNA, 1× specific buffer for reverse transcriptase, 1 mM dNTPs, 10 µM direct primer specific for the dengue virus (DV1-5′-GGRACKTCAGGWTCTCC-3′) and 1 µL reverse transcriptase (Fermentas, Vilnius, Lithuania), as well as incubation at 42 °C for 1 h and enzyme inactivation at 94 °C for 10 min. The following was used for dengue virus amplification: 3 µL cDNA, 1× buffer, 2.0 mM MgCI_2_, 0.2 mM dNTPs, 0.2 µM of each reverse primer specific for the four virus serotypes (DSP1-5′-AGTTTCTTTTCCTAAACACCTCG-3′, DSP2-5′-CCGGTGTGCTCRGCYCTGAT-3′, DSP3-5′-TTAGAGTYCTTAAGCGTCTCTTG-3′ and DSP4-5′-CCTGGTTGATGACAAAAGTCTTG-3), and 0.5 U taq polymerase. PCR amplification was performed at an initial temperature of 95 °C for 2 min, followed by 35 cycles at 95 °C for 30 s, 55 °C for 1 min, and 72 °C for 40 s, with a final extension at 72 °C for 3 min. The size of the NS3 gene fragments obtained was 169 pb for DENV-1, 362 pb for DENV-2, 265 pb for DENV-3 and 426 pb for DENV-4. The protocol used by [25] was applied to detect Zika virus. For chikungunya amplification, the protocol proposed by [26] was used.

Amplification products were analyzed in 2% agarose gels dyed with ethidium bromide and observed under ultraviolet light. For each reaction, extraction and amplification negative controls were included, as well as positive controls comprising RNA of Zika and chikungunya viruses, and the four serotypes of dengue virus obtained from supernatant of infected cells. To confirm pool positivity, 15% of positive samples were randomly selected for bidirectional sequencing at Macrogen (Seoul, Korea).

### 2.4. Epidemiological Data

The number of cases of dengue and Zika in the city of Medellín was obtained from the Colombian public health surveillance system (SIVIGILA), and the accumulated values per trimester were used for the analyses carried out in this study.

### 2.5. Data Analysis

Traditional *Aedes* indices of container (CI), housing (HI), Breteau (BI), and adult mosquito infestation (AI) were calculated following the estimations recommended by the World Health Organization (TDR/WHO 2009). Infection rates (IR) were estimated using the maximum likelihood estimator (MLE) method for differently sized pools [27] through the pooledBin function implemented in the binGroup statistical package of R.

The parameter used to estimate IRs was “bias-corrected MLE” with “skewness-corrected confidence Interval”, as has been described previously in a study testing arboviruses in Colombia [28]. IRs without virus distinction were individually estimated for each mosquito species (*Ae. aegypti* and *Ae. albopictus*), and for both pooled species. IRs were estimated for each dengue serotype (DENV-1, DENV-2, DENV-3, and DENV-4), and Zika virus.

Following Shapiro–Wilk tests for normality of the data, the relationships between IRs and some epidemiological (reported dengue and Zika cases), entomological (*Aedes* indices), and climate variables were explored using Spearman’s correlation analysis. The climate variables included mean temperature (MEANT), maximum absolute temperature (MAXABST), minimum absolute temperature (MINABST), average daily maximum temperatures (AVMAXT), average daily minimum temperatures (AVMINT), maximum daily difference in temperature (MAXDIFT), minimum daily difference in temperature (MINDIFT), average daily difference in temperature (AVDIFT), total precipitation (PRECI), maximum precipitation in 24 h (MAXPRECI), number of days with precipitation (PRECIDAYS), and relative humidity (RH). Epidemiologic data were provided by SSM, whereas monthly climate data were obtained from the Hydrology, Meteorology and Environmental Studies Institute (IDEAM, by its Spanish acronym) of Colombia. Correlations between entomological, epidemiological, and climate variables were analyzed at lags of 0, 1, and 3 trimesters. All analyses were performed in R.

## 3. Results

### 3.1. Mosquito Collections

During the three years of this study, 2362 inspections were performed in schools, and *Aedes* spp. adult mosquitoes were captured in 43.3% of institutions. The percentage of institutions where adult *Aedes* spp. were found varied each trimester with no clear seasonal pattern between years: the highest rate of adult *Aedes* infestation in schools was trimester IV of 2017 (55.16%) and trimester I of 2016 was the lowest (14.29%) (Table 1).

**Figure 2:**
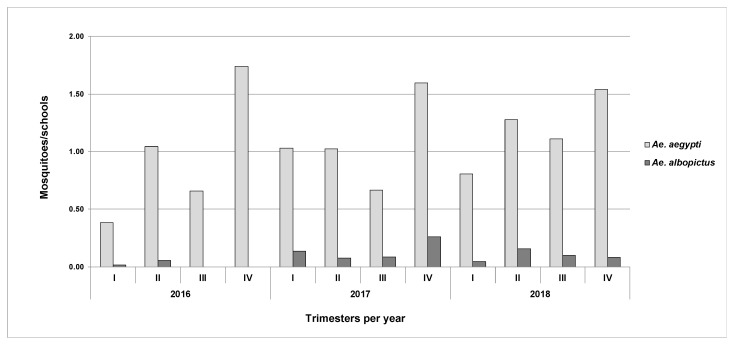
Average number of *Aedes* spp. mosquitoes captured in schools in Medellín between 2016 and 2018.

Overall, 2959 *Aedes* mosquitoes (1265 females, 1694 males) were captured, 91% of which were *Ae. aegypti*. *Aedes aegypti* were collected during all sampling periods, while *Ae. albopictus* were not (Figure 2).

### 3.2. Larval Habitats and Traditional Aedes Indices

Of the 2362 schools inspected, 15% had water-holding containers with immature forms of *Aedes* spp. A total of 8692 containers that were suitable for *Aedes* spp. growth were inspected (and removed in most cases), 8.3% of which tested positive for the presence of mosquito larvae or pupae. Similar to what was observed for adult mosquitoes, the percentage of institutions with containers positive for immature mosquitoes varied by trimester with no clear pattern by trimester between years (Table 2).

According to the PAHO risk parameters (1995), all *Aedes* indices obtained were at a medium risk level. The highest BI and HI levels were recorded in trimester III of 2016, and the highest CI level was recorded in trimester IV of the same year. However, trimester I of 2016 had lower BI and CI values and trimester II of 2018 had a lower HI value (Table 3).

The AI index fluctuated over the study period without a clear pattern. The highest values were recorded in trimesters IV of 2017 (55.16%) and II of 2018 (51.64%), whereas the lowest values were recorded in trimesters I of 2016 (14.29%) and III of 2017 (30.15%) (Table 3).

### 3.3. Molecular Arbovirus Detection in Mosquitoes

Captured mosquitos were divided into 883 pools; 20% of them were positive for dengue in 2016, 6.92% in 2017, and 2.60% in 2018. For ZIKV 9.49% were positive in 2017 and 7.80% in 2018. Chikungunya was not detected in any of the mosquitoes processed during the study period. Across the entire study period, a higher percent of *Ae. aegypti* pools were positive than *Ae. albopictus* pools. Some *Ae. aegypti* pools presented simultaneous DENV/ZIKV infection (Table 4).

### 3.4. Infection Rates

**Figure 3:**
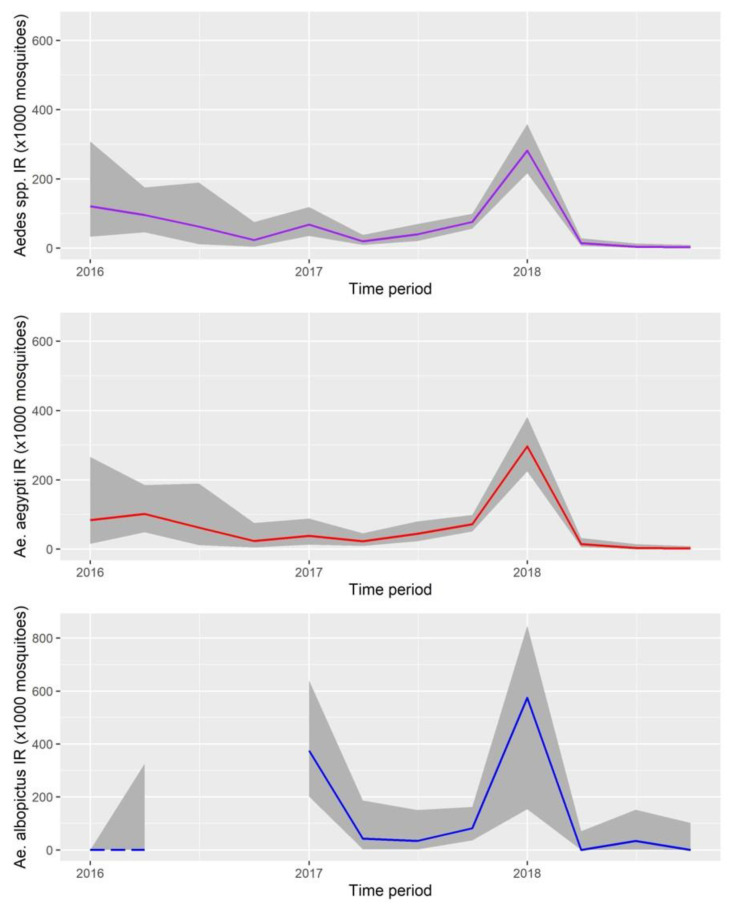
Infections rates without virus distinction with their respective 95% confidence A: IRs species for both pooled. B. IRs for *Ae. aegypti.* C. IRs for *Ae. Albopictus*, discontinuous line and space in year 2016 refers to lack of statistical meaning by analyzing only one pool for each of the two first periods.

Cumulative IRs for *Aedes* spp. varied each trimester, ranging from 2.93 (trimester IV of 2018) to 282.03 (trimester I of 2018). For *Ae. aegypti*, IRs ranged from 1.75 (trimester IV of 2018) to 296.29 (trimester I of 2018). These rates were higher for *Ae. albopictus* because of the low number of mosquitoes (259) compared to *Ae. aegypti* (2692), which resulted in IRs ranging from 0 in most trimesters to 573.98 in trimester I of 2018 (Figure 3).

**Figure 4:**
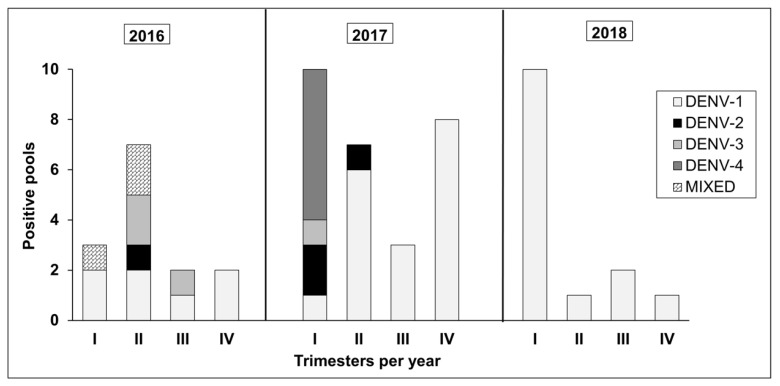
Distribution of dengue serotypes over time in *Aedes* spp. captured in schools inspected between 2016 and 2018 in Medellín, Colombia.

During 2016 and the beginning of 2017, all four DENV serotypes were found; during this same period, Medellín recorded the highest levels of dengue transmission [4]. DENV-1 was the only serotype detected after trimester III of 2017. A single specimen was found to be coinfected with both DENV-2 and DENV-4 (Figure 4). The other mixed infections pools were grouped by several specimens.

**Figure 5:**
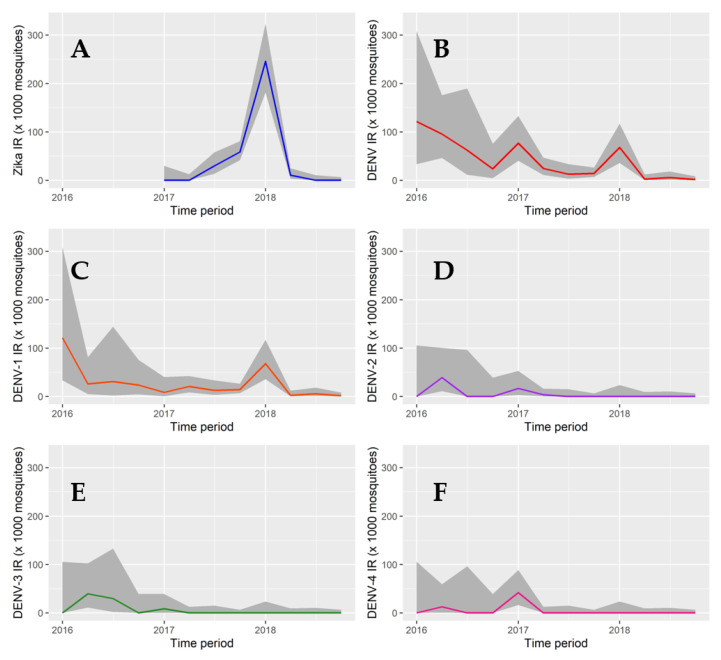
Arbovirus infection rates in mosquitoes captured in schools between 2016 and 2018. The 95% confidence interval is colored in gray. (**A**) Zika virus. (**B**) Dengue virus (all serotyped combined). (**C**) Dengue serotype 1 (DENV-1). (**D**) Dengue serotype 2 (DENV-2). (**E**) Dengue serotype 3 (DENV-3), and (**F**) Dengue serotype 4 (DENV-4).

Changes in IRs for the different serotypes were also observed over time. DENV-1 IR was registered through the entire study and consistently was found at the highest values. The remaining serotypes had similar IR patterns over time (Figure 5); 45% of pools were positive for Zika in trimester I of 2018; this period had the highest infection rate which is also reflected in the IRs over time (Figure 5).

### 3.5. Relationship between Infection Rates and Epidemiological, Entomological, and Climate Variables

We found a significant relationship between *Ae. aegypti* and *Aedes* spp. IRs and number of human dengue cases (Table 5), but no significant relationship was observed between *Ae. albopictus* IRs and disease cases (data are not shown).

For *Aedes container* indices, no statistically significant correlation was found between any entomological indicator and arbovirus infection rates, and no statistical correlation was found for the Lag periods considered. No correlation was found between adult index and these parameters (Table 6).

Table 7 shows the relationship between climate variables and IRs for *Ae. aegypti* and *Ae. albopictus*. Interestingly, results were inconsistent. A positive correlation between minimum absolute temperature, average daily maximum temperatures, minimum daily difference in temperature, average daily minimum temperatures and *Ae. aegypti* IRs was observed; however, it had a negative correlation with precipitation. On the other hand, the mean temperature, average daily maximum temperatures, minimum daily difference in temperature had a negative correlation with *Ae. albopictus* IRs, while relative humidity had a positive correlation.

## 4. Discussion

Traditionally, it has been assumed that dwellings are the main site of dengue transmission given the endophilic and anthropophilic behavior of *Ae. aegypti*. Therefore, most studies measuring vector abundance and routine control have focused on homes [20,29,30,31]. However, because few studies have assessed the risk for transmission in areas other than dwellings that are also highly trafficked and known to have mosquitoes, it is not clear if non-residential locations such as schools are also important places of arboviral transmission [29]. Mosquito abundance in schools has already been recorded in some countries such as Mexico [32], Brazil [33], Thailand [34,35], Peru [14], and Colombia [17,36], but measuring mosquito abundance is inadequate to accurately assess arboviral disease transmission risk. As far as the authors are aware, this is the first study to explore the rate of arbovirus infection in mosquitoes in schools and analyze the relationship between abundance indices, infection rates, and cases.

Our results showed that both immature and adult forms of *Aedes* spp. mosquitoes were detected in a high percentage of schools (43.27%). This is not surprising given the anthropophilic behavior of these vectors. The student population spends a significant amount of time at school, and therefore they may be important transmission foci for arboviruses. A high dengue incidence in student populations has been extensively documented in countries such as Nicaragua [37], Venezuela [2], Thailand [35], Vietnam [38], and even already in Colombia [36,39]. Furthermore, the incorporation of schools into routine vector control programs has proved to be effective in decreasing the presence of both vectors and disease [40]. Thus, they should be key sites for interventions aimed at arbovirus prevention. The presence of *Ae. albopictus* inside schools was a relevant finding, which can be explained by the rapid and aggressive reproduction of this mosquito. Although *Ae. aegypti* is the main vector in Medellín, the continuous increase of *Ae. albopictus* abundancy, its presences across at least 70% of the city neighborhoods [41], and the possibility this species may not be well controlled contribute to increase its role as a vector, not only in Medellín, but also in other cities where it is distributed [42].

Containers with *Aedes* spp. were reported in 15.5% of inspections, although the entomological indices for the periods assessed had a medium risk level according to PAHO parameters (1995). Even during the 2016 epidemic, these values were not higher than the other years in the schools. Our study showed no correlation between *Aedes* indices and dengue infection rates in mosquitoes. The use of *Aedes* indices as risk indicators for dengue transmission has been discussed by different researchers because several studies have reported no association between them and the amount of disease cases [18,27,43]. Some of the weaknesses of these indices are that they do not consider adult mosquito productivity, and most searches are performed on visible containers, thus leaving cryptic breeding containers unattended. Consequently, these indices may not provide full and accurate information for a correct estimation of the transmission risk [43], leading to a defective representation of the actual number of vectors. However, these indices are still being used and required by government agencies in several countries.

Detection of arboviruses in field mosquitoes has been suggested as effective tool to identify outbreaks risk [19].Our results evidenced natural infection of *Aedes aegypti* and *Ae. albopictus* species with dengue virus in the schools of Medellín. Natural infection of *Ae. albopictus* with dengue and Zika viruses has already been reported [9,42,44]; however, its role as an arbovirus vector in the country should be further clarified, especially considering the results in our study. The low amount of *Ae. albopictus* as opposed to *Ae. aegypti* is attributed to the biology of this species, which is less anthropophilic than *Ae. aegypti* [45]. However, the high infection rates observed for *Ae. albopictus* can be explained by the low number of pools evaluated, which results in extensively varied infection rates and less solid statistical analysis. Another aspect that could contribute to this low number of captures was the method implemented; the efficiency of the sampling with oral aspirator is affected by the time spent for sampling and the ability of the person implementing the capture. These variables can cause the number of captures to be low, leading to an inaccurate estimate of infection rates. Consequently, further studies are required given the epidemiological relevance of this species in schools and the significant role it plays in arbovirus transmission in other countries [46,47].

A study performed in schools of Merida (Mexico) showed that 16.1% of the pools assessed were positive for dengue [32], whereas a higher percentage (20%) was observed in this study for 2016. However, this number decreased in 2017 and 2018. Our results may be explained by the fact that a large dengue epidemic was recorded in Medellín in 2016, in which all four serotypes circulated [20]. This is the first study the authors are aware of reporting mosquitoes infected with ZIKV in schools. Other studies [48] have shown natural infection in mosquitoes captured in dwellings, with a positivity percentage similar to what was observed in our study (10% in 2017 and 8% in 2018).

We analyzed which variables influence infection rates, and the results in our study suggested an inversely proportional relationship between precipitation and infection rates, suggesting that warmer and drier periods favor dengue transmission. However, there are divergent results regarding the relationship between precipitation and the disease, e.g., certain studies have shown a positive correlation explained by the existence of vector breeding sites, which could enhance transmission [49]. However, other studies have shown a negative correlation with dengue incidence [50]. We also found a high number of significant correlations with infection rates for *Ae. aegypti*. These associations were closely related with dengue and Zika cases reported in Medellín, which suggests that *Ae. aegypti* is mainly responsible for dengue and Zika viruses transmission in the city or, at least, in schools. Similar results were found by [32] in schools of Merida.

Additionally, we found that temperature variables have a positive effect in arbovirus infection rate, the association between temperature and arbovirus replication and transmission had previously been described [51,52,53], particularly in the municipality of Bello, which shares urban areas with Medellín city [27]. Moreover, it has been reported that an increase in environmental temperature raises the onset of dengue in cities with a mean temperature like the one registered for Medellín [28]. Accordingly, the case increase observed in 2016 could have been attributed to a general temperature increase observed during that year. During these periods, *Ae. albopictus* mosquitoes showed infection rates of zero, which could account for the negative coefficient correlations observed in our study for that species.

## 5. Conclusions

In conclusion, our results evidence the importance of incorporating schools into arbovirus surveillance programs to achieve a higher impact on arbovirus prevention and control activities. Additionally, detection of arbovirus-infected mosquitoes raises an early alarm about the potential risk of transmission in these sites, especially because students, professors and any people working in these places are exposed to infected mosquito bites most of the day. Community participation is essential to control this type of diseases, especially school participation, to eliminate breeding containers inside schools and reduce mosquito populations and consequently reduce the risk of being bitten by an infected female vector.

## Figures and Tables

**Table 1:** Percentage of schools with *Aedes* spp. mosquitoes observed between 2016 and 2018. The total amount of inspected institutions is included in parentheses.

Year	Trimester				Total
	I	II	III	IV	
2016	14.3% (70)	32.9% (70)	32.0% (50)	50.0% (50)	30.8% (240)
2017	49.0% (102)	49.4% (265)	30.1% (325)	55.2% (310)	44.9% (1002)
2018	38.1% (181)	51.6% (275)	40.0% (295)	45.8% (369)	44.5% (1120)

**Table 2:** Percentage of schools with positive containers registered between 2016 and 2018 in Medellín. The total amount of institutions inspected is included in parentheses.

Year	Trimester				Total
	I	II	III	IV	
2016	12.9% (70)	17.1% (70)	20.0% (50)	18.0% (50)	16.7% (240)
2017	17.6% (102)	19.2% (265)	16.6% (325)	17.1% (310)	17.6% (1002)
2018	13.3% (181)	18.5% (275)	10.2% (295)	11.9% (369)	13.3% (1120)

**Table 3:** Traditional *Aedes* indices estimated for schools inspected between 2016 and 2018 in Medellín, Colombia.

Year	Trimester	Schools Inspected	BI	CI	HI	AI	Total Containers
2016	I	70	15.71	4.12	12.86	14.29	267
	II	70	17.14	6.06	17.14	32.86	198
	III	50	48.00	12.44	20.00	32.00	193
	IV	50	46.00	14.74	18.00	50.00	156
2017	I	102	32.35	6.27	17.65	49.02	526
	II	265	46.79	9.05	19.25	49.43	1370
	III	325	33.23	10.14	16.62	30.15	1065
	IV	310	47.10	12.64	17.10	55.16	1155
2018	I	181	22.65	8.97	13.26	38.12	457
	II	275	28.73	6.30	18.55	51.64	1253
	III	295	17.29	6.08	10.17	40.00	839
	IV	369	19.24	5.85	11.92	45.80	1213

BI: Breteau Index, CI: Container Index, HI: House Index; AI: Adult Index.

**Table 4:** Detection of dengue and Zika viruses in *Ae. aegypti* and *Ae. albopictus* collected in schools of Medellín, Colombia, between 2016 and 2018. NE: Non-evaluated.

Year	Species	No. of Pools Tested	No. of DENV-Positive Pools	No. of ZIKV-Positive Pools	No. of ZIKV + DENV-Positive Pools
2016	*Ae. aegypti*	68	13	NE	NE
	*Ae. albopictus*	2	1	NE	NE
2017	*Ae. aegypti*	336	19	31	1
	*Ae. albopictus*	54	8	6	0
2018	*Ae. aegypti*	373	10	29	3
	*Ae. albopictus*	50	1	4	0

**Table 5:** Statistically significant correlations at 95% confidence between vectors IRs and epidemiological (reported cases) and virological (pathogen IRs) variables. No significant correlations were recorded for *Ae. albopictus* species.

Variable	Lag	*Ae. aegypti*		Both Species	
		Rho	*p* Value	Rho	*p* Value
Dengue cases without warning signs	+2	0.6433	0.0279	0.6713	0.0204
Total cases of dengue	+2	0.6433	0.0279	0.6713	0.0204
Zika cases	+1	0.5958	0.0409	0.6171	0.0325

**Table 6:** Correlation between IRs and *Aedes* indices for *Ae. aegypti, Ae. albopictus,* and both species combined.

	*Ae. aegypti*		*Ae. albopictus*		Both Species	
	Rho	*p* Value	Rho	*p* Value	Rho	*p* Value
BI	−0.0629	0.8517	0.2538	0.4260	−0.1119	0.7328
CI	0.0839	0.8002	0.2463	0.4402	0.007	0.9912
HI	0.0280	0.9387	−0.1082	0.7377	−0.007	0.9912
AI	−0.4266	0.1689	0.1941	0.5456	−0.3706	0.2367

**Table 7:** Correlations at 95% confidence between vector IRs and climate variables.

Variable	Lag	*Ae. aegypti*		*Ae. albopictus*	
		Rho	*p* Value	Rho	*p* Value
MEANT	−1	NS	NS	−0.7949	0.0019
MINABST	−2	0.6325	0.0273	NS	NS
AVMAXT	−1	NS	NS	−0.6308	0.0279
	−2	0.6084	0.04	NS	NS
AVMINT	0	0.6619	0.019	NS	NS
	−1	0.7565	0.0044	NS	NS
MINDIFT	−1	NS	NS	−0.7166	0.008
MAXPRECI	−1	−0.6363	0.0301	NS	NS
RH	−1	NS	NS	0.6419	0.0244

NS: Non-significant statistical relationship. MINABST: minimum absolute temperature; AVMAXT: average daily maximum temperatures; AVMINT: average daily minimum temperatures; MINDIFT: minimum daily difference in temperature; MAXPRECI: maximum precipitation in 24 h; RH: relative humidity.
